# Transcription Factor *SATB2* Regulates Skeletal Muscle Cell Proliferation and Migration via *HDAC4* in Pigs

**DOI:** 10.3390/genes15010065

**Published:** 2024-01-02

**Authors:** Fanqinyu Li, Chao Yan, Yilong Yao, Yalan Yang, Yanwen Liu, Danyang Fan, Junxing Zhao, Zhonglin Tang

**Affiliations:** 1 College of Animal Science, Shanxi Agricultural University, Jinzhong 030801, China; lfqyyx@126.com; 2 Key Laboratory of Livestock and Poultry Multi-Omics of MARA, Agricultural Genomics Institute at Shenzhen, Chinese Academy of Agricultural Sciences, Shenzhen 518124, China; yanchao@caas.cn (C.Y.); yangyalan@caas.cn (Y.Y.); liuyanwen2021666@163.com (Y.L.); 18339969590@163.com (D.F.); 3 Kunpeng Institute of Modern Agriculture at Foshan, Chinese Academy of Agricultural Sciences, Foshan 528226, China; yaoyilong@caas.cn; 4 Shenzhen Branch, Guangdong Laboratory for Lingnan Modern Agriculture, Agricultural Genomics Institute at Shenzhen, Chinese Academy of Agricultural Sciences, Shenzhen 518124, China

**Keywords:** pig, skeletal muscle, *SATB2*, transcription factor, *HDAC4*

## Abstract

Skeletal muscle development remarkably affects meat production and growth rate, regulated by complex regulatory mechanisms in pigs. Specific AT sequence-binding protein 2 (*SATB2*) is a classic transcription factor and chromatin organizer, which holds a profound effect in the regulation of chromatin remodeling. However, the regulation role of *SATB2* concerning skeletal muscle cell fate through chromatin remodeling in pigs remains largely unknown. Here, we observed that *SATB2* was expressed higher in the lean-type compared to the obese-type pigs, which also enriched the pathways of skeletal muscle development, chromatin organization, and histone modification. Functionally, knockdown *SATB2* led to decreases in the proliferation and migration markers at the mRNA and protein expression levels, respectively, while overexpression *SATB2* had the opposite effects. Further, we found histone deacetylase 4 (*HDAC4*) was a key downstream target gene of *SATB2* related to chromatin remodeling. The binding relationship between *SATB2* and *HDAC4* was confirmed by a dual-luciferase reporter system and ChIP-qPCR analysis. Besides, we revealed that *HDAC4* promoted the skeletal muscle cell proliferation and migration at the mRNA and protein expression levels, respectively. In conclusion, our study indicates that transcription factor *SATB2* binding to *HDAC4* positively contributes to skeletal muscle cell proliferation and migration, which might mediate the chromatin remodeling to influence myogenesis in pigs. This study develops a novel insight into understanding the molecular regulatory mechanism of myogenesis, and provides a promising gene for genetic breeding in pigs.

## 1. Introduction

As the largest organ in pigs, skeletal muscle is a remarkable protein resource concerning meat production, and also serves as an essential biomedical organ for studying human skeletal muscle growth, development, and health [1,2]. Indeed, myogenesis is a vital part of skeletal muscle development, which is involved in myoblast proliferation, migration, differentiation, fusion, and so on [3]. It is universally acknowledged that myogenesis is a highly precise, tightly coordinated, and well-organized multi-step biological event [4]. The process of myogenesis is maintained and regulated not only by a series of muscle-specific transcription factors, such as *Myf5*, *MyoD*, and *MyoG*, but it is also modulated by diverse epigenetic mechanisms [5]. Particularly, epigenetic regulations exert critical roles in myogenesis and skeletal muscle development, mainly including methylation and demethylation, chromatin remodeling, and post-translational modifications of histones [6,7,8]. Thus, it is of great importance to dissect the epigenetic regulatory network of myogenesis for pig meat production and human skeletal muscle health.

Chromatin remodeling, as an important part of epigenetic regulations, has been a focus of epigenetic modification for skeletal muscle satellite cells [9,10]. As studied, it is closely associated with different chromatin remodeling complexes [11]. For example, the ATP-dependent chromatin remodeling complex SWI/SNF promotes myoblasts differentiation through the mediation of *MyoD* [10]. Besides, chromatin remodeling is often closely associated with histone modifications [12]. That is, different histone post-translational modifications can affect chromatin formation and openness, and facilitate regulation of skeletal muscle growth and development, such as chromatin remodeling factor *HDAC1* and *HDAC4* [13]. Of note, to enable the accessibility of transcription factors to their target sequences, the chromatin occurred to remodel into a less compacted structure [14]. Therefore, chromatin remodeling exerts vital roles for gene expression and myogenesis programs.

Specific AT sequence-binding protein 2 (*SATB2*) is a member of an enriched family of AT sequence-binding proteins and is defined as a classic transcription factor and chromatin organizer [15]. As reported, *SATB2* as a nuclear matrix-associated transcription factor plays an active role in the modulation of chromatin remodeling and gene transcription [15,16]. For example, in cortical neuronal cells, *SATB2* can alter the location and structure of chromatin orchestrated by *HDAC1* and metastasis-related protein 2, providing direct evidence for the regulation of chromatin remodeling [17]. *SATB2* also modulates neuronal migration via the employment of chromatin remodeling factors to the Ctip2 locus [18]. In addition, *SATB2* can regulate chromatin structure changes, in terms of regulating the alterations of the A/B compartment, topological association domain [19] and loop structure in the three-dimensional space of chromatin, and promote the differentiation of skeletal muscle cells in mice [20]. Noticeably, *SATB2* can interact with chromatin-remodeling molecules medicating chromatin organization in the differentiation of cortical neurons [18]. However, *SATB2* regulating cell fate of porcine skeletal muscle through chromatin remodeling remains greatly unclear.

This study was aimed to elucidate the regulatory molecular mechanism of *SATB2* in porcine skeletal muscle concerning chromatin remodeling. Firstly, we characterized *SATB2* by 27 growth and development time points of transcriptome profiling of skeletal muscle. We investigated the functionality of *SATB2* via knockdown and overexpression concerning the skeletal muscle developmental process. We also analyzed the target genes of SATB2 and then explored its genetic mechanisms concerning chromatin remodeling to regulate myogenesis programs. Our study reveals the function and molecular mechanism of the *SATB2* gene in regulating skeletal muscle cell progress via chromatin remodeling factors. This study will provide a promising gene and theoretical basis for the advancement of meat production performance and molecular breeding in pigs.

## 2. Materials and Methods

### 2.1. Cell Culture and Transfection

Porcine skeletal muscle cells (BIOSPECIES-0017a) were purchased from Guangzhou Suyan Biotechnology Co., Ltd., Guangdong, China. 293T cells were collected from the American Type Culture Collection (ATCC, Manassas, VA, USA). All experimental cells in our study were cultured in Dulbecco’s Modified Eagle’s Medium (DMEM, Gibco, Grand Island, NY, USA) containing 10% fetal bovine serum (FBS, Gibco, Grand Island, NY, USA) and 1% penicillin-streptomycin (PS, Thermo Scientific, Waltham, MA, USA), and maintained and cultured in a humidified incubator with 37 °C and 5% CO_2_. The transfection reagent was jetprime (Polyplus-transfection, llkirch, France) and operated according to the instructions. 

### 2.2. Lentivirus Packaging

As studied, lentiviral infection is performed to deliver target transgenes to 293T cells with the robust expression of stable cell lines [21]. In this study, we developed the lentivirus in 293T cells using three vectors: 2.1 μg pLV3-CMV-MCS-3×FLAG-CopGFP-Puro or pLV3-CMV-*SATB2* (pig)-3×FLAG-CopGFP-Puro, 1.4 μg psPAX2 (MiaoLingBio, Wuhan, China), and 0.7 μg PDM2.G (Addgene, Watertown, MA, USA). For lentivirus infection of myoblasts, 1 mL virus and 1 μg polybrene were jointly added to each milliliter of DMEM and then replaced with fresh DMEM after the treatment of 24 h. After 60 h, the virus was collected, and stored at −80 °C for further experiment used.

### 2.3. Primers and Oligonucleotides

In order to explore the effects of overexpression *SATB2*, the overexpression vectors pLV3-CMV-MCS×FLAG-CopGFP-Puro as normal control (*SATB2*-NC), and pLV3-CMV-*SATB2* (pig)-3×FLAG-CopGFP-Puro as overexpression (*SATB2*-OE) were obtained from MiaoLingBio (Wuhan, China). Meanwhile, the tools of decreased specific gene expression with siRNA, including siRNA-*SATB2* (CCAAATCCACCACAGTACT) and siRNA-*HDAC4* (GCTCAAGAACAAGGAGAAA) as knockdown oligonucleotides and siRNA-NC were obtained from RiboBio (Guangzhou, China). 

### 2.4. 5-Ethynyl-2′-Deoxyuridine Assay

Proliferation cells were measured using EdU staining described previously by a study [22]. In brief, porcine skeletal muscle cells were seeded in 12-well plates with three repetitions. At 50% confluence, cells were transfected with siRNA-*SATB2* or siRNA-*HDAC4*, and siRNA-NC. After siRNA transfection for 48 h, myoblasts were detected by EdU staining and exposed to 50 μM EdU (Beyotime, Beijing, China) for 2 h at 37 °C. Subsequently, processed myoblasts were fixed in 4% paraformaldehyde for 30 min, and washed three times with PBS, and permeabilized with 0.5% Triton X-100. A solution containing EdU (Apollo Reaction Cocktail, Beyotime, Beijing, China) was added to the processed myoblasts and incubated for 30 min at room temperature. The Hoechst staining solution was then added and incubated for another 30 min at room temperature. The number of EdU-stained myoblasts was visualized using a fluorescence microscope (DMi8, Leica, Wetzlar, Germany) to capture three randomly selected fields.

### 2.5. Real-Time Quantitative PCR (RT-qPCR)

The gene expressions were detected by RT-qPCR referred to in our previous study [8]. The myoblasts originated from pig leg muscle processed in the 12-well plates were washed by PBS, and digested by Trizol (Invitrogen, Shanghai, China). The RNA was extracted from myoblast and the quality and quantity was checked. The qualified RNA was used for further study. According to the instructions, HiScript III 1st Strand cDNA Synthesis Kits (R312-01, Vazyme, Nanjing, China) were used for cDNA reverse transcription synthesis, respectively. In addition, RT-qPCR was performed in a total reaction volume of 10 μL, including 5 μL 2 × SYBR Master Mix (Vazyme, Nanjing, China), 0.2 μL forward and reverse primers, 1 μL cDNA template, and 3.6 μL sterile water. The reaction conditions were 95 °C for 30 s, then 95 °C for 10 s, and 60 °C for 30 s for 40 amplification cycles, and 72 °C for 30 s. The reference gene *β-actin* was being widely used and considered as an endogenous housekeeping gene [23,24]. The relative expression levels of mRNA were analyzed by the 2^−∆∆CT^ method. The sequences of primers were synthesized by Sangon Biotech, Shanghai, China and shown in Table 1.

### 2.6. Western Blot

Western blot was performed as previously described in [25]. Briefly, the protein was extracted from myoblasts by protein lysate. The lysate mainly consisted of RIPA buffer (Thermo Scientific, Waltham, MA, USA), phosphorylase inhibitor (Roche 5892791001, Basel, Switzerland), and protease inhibitor (Roche 04693132001, Basel, Switzerland). The original protein concentrations were tested and measured by the Enhanced BCA Protein Assay Kit (Beyotime, Beijing, China). Sodium dodecyl sulfate (SDS, CWBIO, Beijing, China) was mixed with prepared protein, and denatured at 100 °C for 20 min. The prepared protein samples were separated by electrophoresis in SDS-polyacrylamide gels (EpiZyme, Shanghai, China). Then, the refabricated adhesives were transferred to the 0.45 μm Hybridization Nitrocellulose Filter membrane (Merck, Rahway, NJ, USA). The primary antibody was incubated overnight at 4 °C. Further, the secondary antibody was incubated for 1.5 h at 4 °C after the membrane had been blocked in 5% skimmed milk for 2 h. Protein bands were exposed to chemiluminescent reagents (Millipore, Bedford, MA, USA) and quantified using Image J (NIH, Bethesda, MD, USA). Primary antibodies SATB2 (81 kDa, 1:1000, Affinity Biosciences DF2962, Liyang, China), KI67 (358 kDa, 1:1000, Bioss bs-23102R, Beijing, China), PCNA (34 kDa, 1:1000, Affinity Biosciences AF0239, Liyang, China), CDK4 (34 kDa, 1:1000, Cell Signaling Technology, 1290S, Danvers, MA, USA), CYCLIND2 (50 kDa, 1:1000, Affinity Biosciences AF5410, Liyang, China), FHL1 (36 kDa, 1:1000, Affinity Biosciences DF13482, Liyang, China) and GAPDH (37 kDa, 1:1000, Abcam ab9482, Cambridge, UK) were diluted by 1× Tween buffer (EpiZyme, Shanghai, China). Secondary antibodies were derived from rabbits and mice (ZSGB-BIO, ZB-2305, Beijing, China). The targeted and referenced proteins were detected using the Gel Doc XR System (Bio-Rad, Hercules, CA, USA) as per the instructions of the manufacturer.

### 2.7. Cell Cycle Assay

After 48 h of transfection, myoblasts were collected and fixed with 70% ethanol at 4 °C for 2 h. Myoblasts were then stained with a solution containing propidium iodide (0.05 mg/mL), RNase A (1 mg/mL), and 0.3% Triton X-100 for 30 min in the darkroom. DNA content (propidium iodide intensity) was measured using a flow cytometer (CytoFLEX, Miami, FL, USA) to detect the proportion of cells in four phases of the myoblast cell cycle. The G1, S, and G2/M phases cell populations were analyzed using ModfitLT 5.0 software (Topsham, ME, USA). A total of 10,000 cells were analyzed per sample. Each assay was independently repeated three times.

### 2.8. Wound Healing Assay

The wound healing assay was performed as previously described in [26]. In brief, porcine skeletal muscle cell lines were inoculated in 6-well plates for 24 h before transfection. After transfection, when the myoblasts fusion reached approximately 90%, the myoblasts were scratched along a straight line with the tip of a 200 μL sterile pipette. Then, pictures were taken with a 40Í microscope (DMi8, Leica, Wetzlar, Germany) at 0 h and 6 h, respectively. The area of the wounds was analyzed using Image J 1.51k software (NIH, Bethesda, MD, USA). Each experiment was independently repeated three times.

### 2.9. Transwell Assay

The transwell assay was conducted according to the reported study [27]. Briefly, after 48 h of transfection, the porcine skeletal muscle cell line was cultured with 1% PS DMEM and then starved for 12 h at 37 °C. After digestion, 1 × 10^5^ myoblasts were seeded into the upper chamber of a 24-well transwell (Corning, New York, NY, USA), and 100 μL serum-free DMEM medium was added. DMEM medium with 10% FBS and 1% PS (500 μL) was then added to the lower chamber of transwell. Then, 6 h later, the transwell upper chamber was removed, washed with PBS, fixed with 4% paraformaldehyde, and stained with DAPI (Beyotime, Beijing, China). Finally, myoblasts migration was observed under a 40Í microscope (DMi8, Leica, Wetzlar, Germany) and statistically analyzed using Image J 1.51k software. Each experiment was independently repeated three times.

### 2.10. Plasmid Construction

To explore the relationship between *SATB2* and *HDAC4*, the wild type (WT) and mutation type seed sequences of *HDAC4* were ligated into pLV3-CMV-MCS×FLAG-CopGFP-Puro (pLV3-*HDAC4*-WT and pLV3-*HDAC4*-MUT) [28]. RT-qPCR analysis of *HDAC4* expression levels in porcine skeletal muscle cells was performed after transfection with 50 nM pLV3-*HDAC4*-WT, pLV3-*HDAC4*-MUT overexpression vectors, and PGL-basic (control) in growth medium. This vector construction was based on Promega dual-luciferase technology, with firefly luciferase (FLUC) used as the primary reporter to monitor mRNA regulation and Renilla luciferase (RLUC) acting as a check reporter for normalization and selection. To determine whether *HDAC4* directly targets *SATB2*, we constructed wild-type *HDAC4* reporter plasmids (pLV3-*HDAC4*-WT) and mutant plasmids by changing the binding sequences of *HDAC4* (pLV3-*HDAC4*-MUT). The mutation sequences of *HDAC4* are listed in Table 2.

### 2.11. Dual-Luciferase Reporter Assay

Dual-luciferase reporter assay provides an efficient means of performing two reporter assays. In accordance with the manufacturer’s instructions, HEK293T cells were expanded to 75~80% confluence in 12-well plates before being co-transfected with 50 nM pLV3-*HDAC4*-WT, pLV3-*HDAC4*-MUT vector, PGL3-basic, and 50 nM *SATB2*-OE using jetprime. Cells were harvested after 24 h, and luciferase activity was evaluated using a dual-luciferase assay system (Promega, Madison, WI, USA).

### 2.12. SATB2-Related Gene Set Enrichment Analysis (GSEA)

To explore the expression and enrichment of *SATB2*, we employed an RNA-seq dataset spanning 27 time points, ranging from embryonic day 33 to postnatal day 180 in Tongcheng and Landrace pigs (NCBI: GSE157045), as well as the transcriptome data in Luchuan and Duroc pigs [2]. In addition, SATB2 single gene GSEA was performed by ranking the correlations between *SATB2* with all genes, and calculating enrichments of the set of genes [29]. GSEA was performed using the “ClusterProfiler” package in R (v4.2.3). 

### 2.13. Chromatin Immunoprecipitation (ChIP)-qPCR Assay

ChIP-qPCR is widely used to test the binding between DNA and protein. ChIP was performed using a ChIP-IT^®^ qPCR Analysis Kit (43513, Active Motif, Shanghai, China) according to the manufacturer’s protocol. Briefly, we conducted each ChIP assay using 1 μg antibodies. The antibodies included SATB2 (sc-81376, Santa Cruz Biotechnology, Santa Cruz, CA, USA), and immunoglobulin G (IgG, #B900610, Proteintech) as the negative control. We conducted RT-qPCR using the retrieved and purified DNA. For qPCR of immunoprecipitated DNA, targeting primers were used to analyze the enrichment levels at *HDAC4* genes. The *HDAC4* primer for ChIP–qPCR was as follows: (Forward: 5′-AGGTTTGCAAATGTCCAGCG-3′, Reverse: 5′-GCAGCACTTGGAGGTCCTAT-3′).

### 2.14. Statistical Analysis

GraphPad Prism 8.0 software was applied to all statistical analyses. The correlation of *SATB2* and *HDAC4* expressions was calculated by Pearson. All of the data were triplicate and are presented as mean ± S.E.M. The *t*-test was adopted to detect differences between groups for statistical significance. *p*-value ≤ 0.05, *p*-value ≤ 0.01 and *p*-value ≤ 0.001 were considered to be differences, significant difference, and extremely significant difference, respectively.

## 3. Results

### 3.1. Expression Pattern of SATB2 in Skeletal Muscle from Obese-Type and Lean-Type Pigs

**Figure 1:**
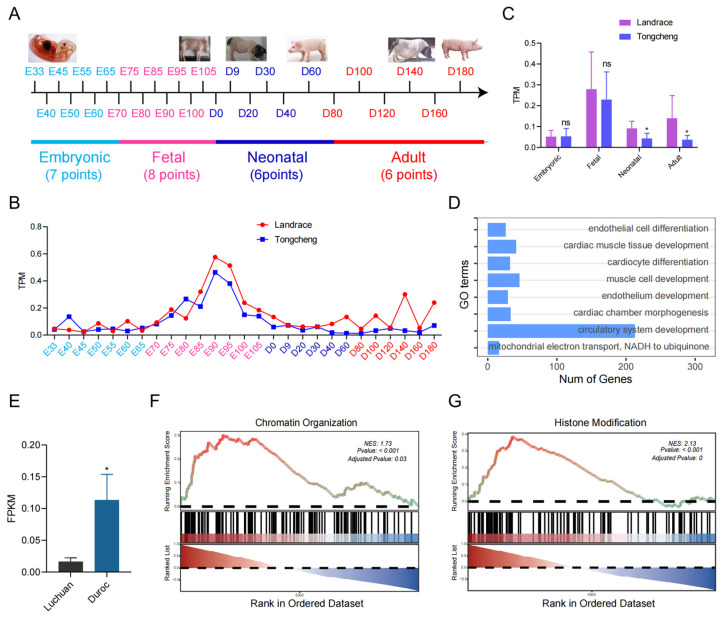
The characteristics of *SATB2* between obese-type and lean-type pigs. (**A**) transcriptome profiling of the skeletal muscle across 27 growth and development time points in Tongcheng (obese-type) and Landrace (lean-type) pigs. (**B**) *SATB2* characterizes various expression patterns among four development phases. (**C**) *SATB2* was expressed higher in the neonatal, and adult phases in the Landrace pig compared to the Tongcheng pig. (**D**) GSEA analysis found that *SATB2* was associated with the GO terms of muscle cell development. (**E**) *SATB2* is higher in the lean-type pig compared to the obese-type pig. *SATB2*-related GSEA analysis showed that *SATB2* was enriched in terms of chromatin organization (**F**) and histone modification (**G**). *Data* are presented as the mean ± S.E.M; * *p* < 0.05, ns (not significant).

According to our previous study, we collected transcriptome profiling of the skeletal muscle across 27 growth and development time points in Tongcheng (obese-type) and Landrace (lean-type) pigs (Figure 1A). In particular, *SATB2* characterizes various expression patterns among the embryonic, fetal, neonatal, and adult development phases between Tongcheng and Landrace pigs (Figure 1B). Further, we found that *SATB2* was expressed higher in the neonatal, and adult phases in the Landrace pig compared to the Tongcheng pig (*p* < 0.05; Figure 1C). To demonstrate the functions of *SATB2*, we performed *SATB2*-related GSEA analysis and found that *SATB2* was associated with the GO terms involved in muscle cell development (Figure 1D). Besides, we collected other skeletal muscle transcriptic data from Luchuan (obese-type) and Duroc (lean-type) pigs, and verified that *SATB2* was higher in the lean-type pig compared to the obese-type pig (*p* < 0.05; Figure 1E). Noticeably, *SATB2*-related GSEA analysis showed that *SATB2* was enriched in the GO terms associated with chromatin organization (NES = 1.73, *p* < 0.001; Figure 1F) and histone modification (NES = 2.13, *p* < 0.001; Figure 1G).

### 3.2. SATB2 Affects the Proliferation of Porcine Skeletal Muscle Cells 

**Figure 2:**
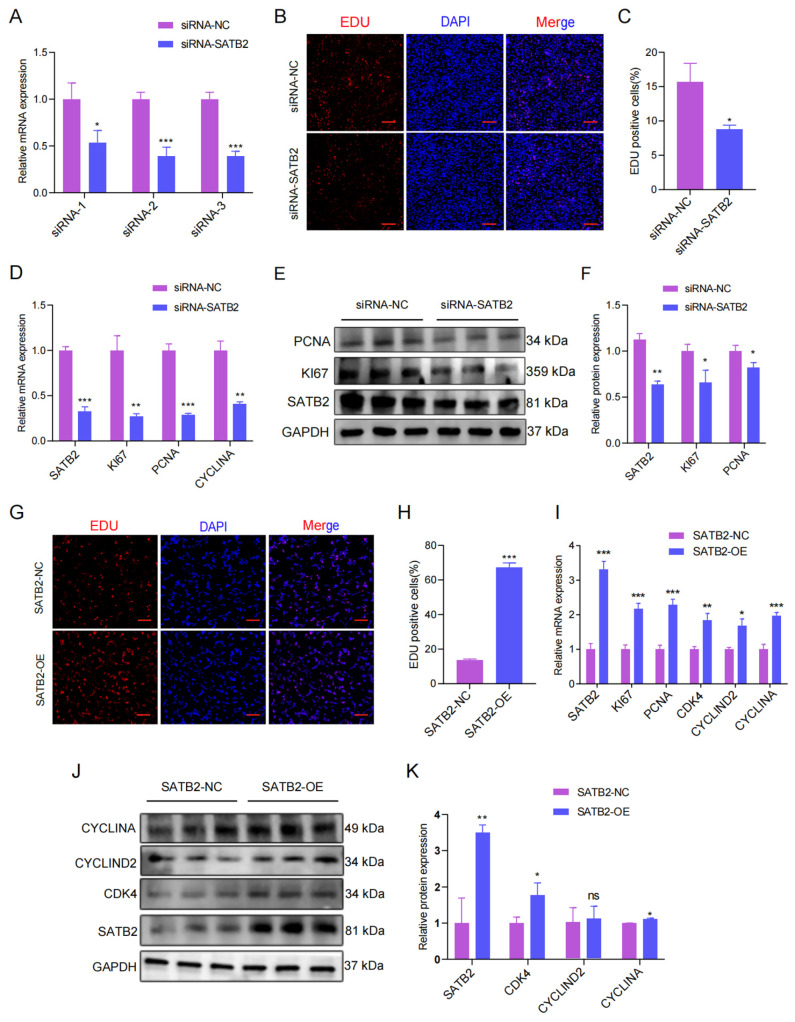
SATB2 enhances the proliferation of porcine skeletal muscle cells. (**A**) Select the best siRNA transfection. (**B**) 48h after transfection, EdU-positive cells were detected, DNA replication cells with EdU staining (red) and nuclei with Hoechst staining (blue). (**C**) The number of EdU positive cells were counted with Image J. (**D**) The mRNA expression of KI67, PCNA, and CYCLINA after the knockdown of SATB2 was analyzed by RT-qPCR. (**E**) Protein expression levels of KI67, PCNA after SATB2 knockdown by Western blot. (**F**) Protein gray-scale values were evaluated by the Image J method. (**G**) The number of positive cells, DNA replication cells with EdU staining (red) and nuclei with Hoechst staining (blue). (**H**) The number of EdU positive cells were counted with Image J. (**I**) The mRNA expression of KI67, PCNA, CDK4, CYCLIND2, and CYCLINA after the overexpression of SATB2 was determined by RT-qPCR. (**J**) Protein levels of KI67, PCNA after overexpression of SATB2. (**K**) Protein gray-scale values were evaluated by the Image J. Data are presented as the mean ± S.E.M from three independent experiments; * *p* < 0.05, ** *p* < 0.01, *** *p* < 0.001, ns (not significant).

Due to cell proliferation being close to skeletal muscle development, we thus explored SATB2 function concerning cell proliferation. To explore the SATB2 functions in the proliferation and migration of porcine myoblasts, we designed siRNA of SATB2, and selected siRNA-2 with the best transfection (*p* < 0.05; Figure 2A). The EdU results indicated that SATB2 knockdown reduced the number of EdU-positive cells (*p* < 0.05; Figure 2B,C). The RT-qPCR and Western blot experiments showed that SATB2 knockdown decreased the mRNA expression of proliferation marker genes (KI67, PCNA, and CYCLINA) (*p* < 0.05; Figure 2D) and the protein level of KI67 and PCNA (*p* < 0.05; Figure 2E,F), respectively. In contrast, overexpression of SATB2 increased the number of EdU-positive cells (*p* < 0.001; Figure 2G,H), upregulated the mRNA expression of skeletal muscle proliferation marker genes (KI67, PCNA, CDK4, CYCLIND2, and CYCLINA) (*p* < 0.05; Figure 2I), and the protein expression levels of CDK4, CYCLIND2, and CYCLINA (*p* < 0.05; Figure 2J,K). Besides, the cell cycle after knockdown SATB2 was different from the control group concerning G1 (66.78%), S (30.68%), and G2/M (2.54%) phase cell populations (Figure S1A). Conversely, the overexpression of SATB2 increased the cell cycle compared to the SATB2-NC (Figure S1B). 

### 3.3. SATB2 Modulates the Migration of Porcine Skeletal Muscle Cells

**Figure 3:**
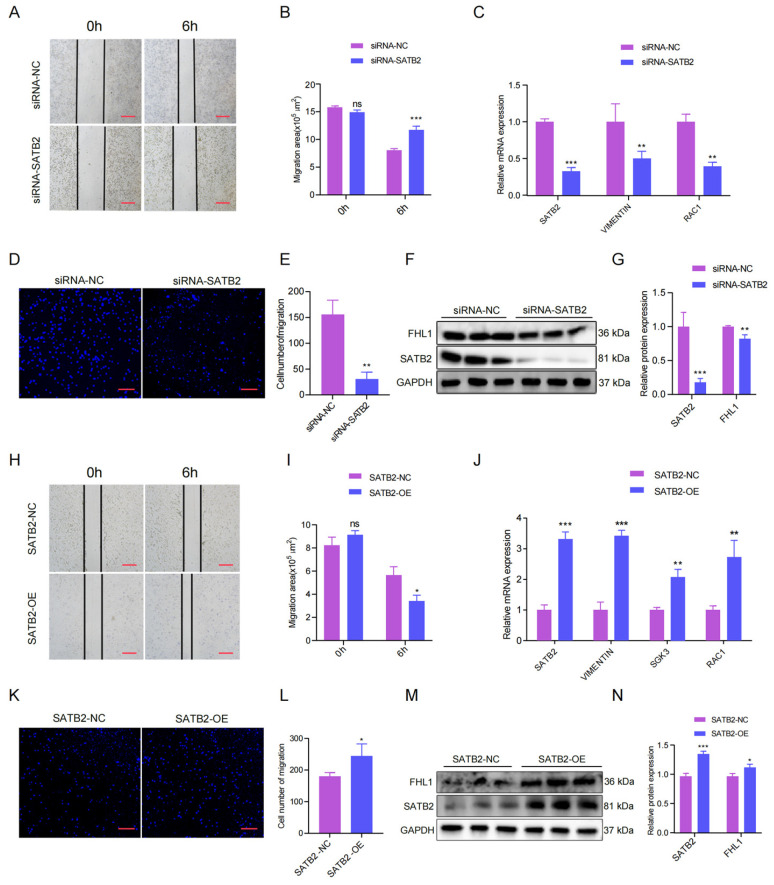
*SATB2* regulates the migration of pig skeletal muscle cells. (**A**) Effect of *SATB2* knockdown on wound healing at 6 h after scratch treatment (40×). (**B**) Changes in the scratch area of pig myoblasts. (**C**) The mRNA expression levels of *RAC1* in *VIMENTIN* after *SATB2* knockdown was measured by RT-qPCR. (**D**) Knockdown of *SATB2* reduced the number of porcine skeletal muscle cells that migrated in the transwell assay (40×). (**E**) Number of pig skeletal muscle cells undergoing migration. (**F**) Protein levels of FHL1 after SATB2 knockdown were measured by Western blot. (**G**) Protein gray values were evaluated by Image J. (**H**) Effect of overexpression of *SATB2* on wound healing at 6 h after scratch treatment (40×). (**I**) Changes in the scratch area of pig skeletal muscle cells. (**J**) The mRNA expression of *VIMENTIN*, *SGK3*, and *RAC1* after *SATB2* overexpression was determined by RT-qPCR. (**K**) Overexpression of *SATB2* increased the number of porcine skeletal muscle cells that migrated in the transwell assay (40×). (**L**) Number of pig skeletal muscle cells undergoing migration. (**M**) Protein levels of FHL1 after overexpression of SATB2 was determined by Western blot. (**N**) Protein gray values were evaluated by Image J. All experiments were independently repeated at least three times and normalized using *GAPDH* or *β-actin*. Data were given as the mean of ± S.E.M. * *p* < 0.05, ** *p* < 0.01, *** *p* < 0.001, ns (not significant).

As reported, SATB2 is associated with skeletal muscle migration concerning skeletal muscle development, which is not reported in pigs. Therefore, we verified the role of SATB2 in the migration of porcine skeletal muscle cell lines. The wound healing assay suggested that SATB2 knockdown inhibited the migration of pig myoblasts (Figure 3A) and the migration area was increased after knockdown SATB2 (*p* < 0.001; Figure 3B). The RT-qPCR analysis showed that the knockdown of SATB2 downregulated migration marker genes (VIMENTIN and RAC1) at the mRNA level in porcine myoblasts (*p* < 0.01; Figure 3C). The transwell migration assay further indicated that knockdown of SATB2 reduced the number of cells migrating (*p* < 0.05; Figure 3D,E). Consistently, the Western blot analysis suggested that the knockdown of SATB2 down-regulated the expression of the migration marker gene (FHL1) in porcine myoblasts (*p* < 0.05; Figure 3F,G). In contrast, overexpression of SATB2 promoted the migration indexes of porcine skeletal muscle cells, including the migration area, migration marker gene, and the number of cells migrating (*p* < 0.05; Figure 3H–N).

### 3.4. HDAC4 Is a Downstream Target of SATB2 Co-Activator

**Figure 4:**
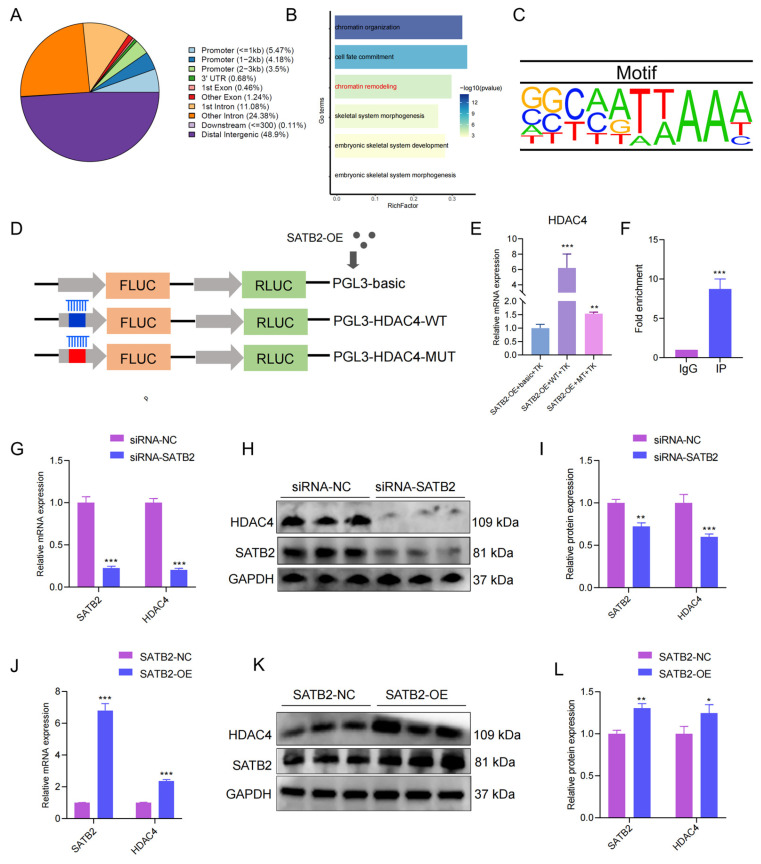
Screening for the *SATB2* downstream target gene *HDAC4*. (**A**) The distribution of peaks for *SATB2* chip-seq. (**B**) The genes close to the peaks enriched in the GO terms. (**C**) Motif of *SATB2*. (**D**) Schematic diagram of the dual luciferase assay. (**E**) Dual luciferase assay validates *SATB2* binding to *HDAC4*. (**F**) ChIP-qPCR assay to validate SATB2 binding to HDAC4. (**G**) RT-qPCR verified that the knockdown of SATB2 was followed by a decrease in HDAC4. (**H**) Protein levels of HDAC4 after SATB2 knockdown were measured by Western blot. (**I**) Protein gray values were evaluated by Image J. (**J**) RT-qPCR verified that overexpression of SATB2 was followed by elevation of HDAC4. (**K**) Protein levels of HDAC4 after overexpression of SATB2. (**L**) Protein gray values were evaluated by Image J. All experiments were repeated at least three times and normalized using GAPDH. Data were given as the mean of ± S.E.M. * *p* < 0.05, ** *p* < 0.01, *** *p* < 0.001.

SATB2, as a transcription factor, could exert its genetic regulation role via a target gene. It has been discovered that SATB2 is homologous and shows high evolutionary conservation between pigs and mice [30]. We collected the public data of SATB2 ChIP-seq of mice (GSE185434) and explored its binding site. The SATB2 binding sites were most distributed in the distal intergenic region (Figure 4A). In addition, we analyzed the nearby genes of peak and found some genes enriched in GO terms for chromatin remodeling (Figure 4B). In the chromatin remodeling, we found a critical gene HDAC4 relating to the histone of chromatin remodeling, binding by SATB2 through AATTAA sequences (Figure 4C). First of all, we successfully mutated the target sequences (Figure S2A). Subsequently, the dual luciferase assay was performed with a vector containing the wild-type sequence (pLV3-HDAC4-WT) or a mutant sequence (pLV3-HDAC4-MUT) to verify the target relationship between SATB2 and HDAC4 (Figure 4D). The luciferase activity of SATB2-OE+WT+TK was significantly higher than the SATB2-OE+basic+TK, while SATB2-OE+MT+TK showed a decrease compared to the SATB2-OE+WT+TK (*p* < 0.001; Figure 4E). Besides, SATB2 ChIP-qPCR was used to further confirm the relationship between SATB2 and HDAC4 (*p* < 0.001; Figure 4F). In addition, we knocked down SATB2 and examined the expression of HDAC4 to further determine the binding relationship. Both RT-qPCR and Western blot analysis showed that HDAC4 expressions were down-regulated under SATB2 knockdown (*p* < 0.001; Figure 4G–I). Oppositely, the overexpression of SATB2 upregulated the HDAC4 expression at mRNA and protein levels (*p* < 0.001; Figure 4J–L). Moreover, the HDAC4 profile was analyzed through transcriptome, (Figure S2B), and it was found that the target gene increased in the adult phase in Landrace compared to the Tongcheng pigs (Figure S2C) and positively correlated with SATB2 (*p* < 0.05, R^2^ = 0.4480; Figure S2D). Meanwhile, the HDAC4 expression was higher in the Duroc pigs (lean-type) than the Luchuan pigs (obese-type) (Figure S2E), although it was not positively correlated with SATB2 (*p* > 0.05, R^2^ = 0.3924; Figure S2F). These data indicated that SATB2 binds HDAC4 to regulate the myogenesis process.

### 3.5. HDAC4 Regulates the Proliferation and Migration of Porcine Skeletal Muscle Cells 

**Figure 5:**
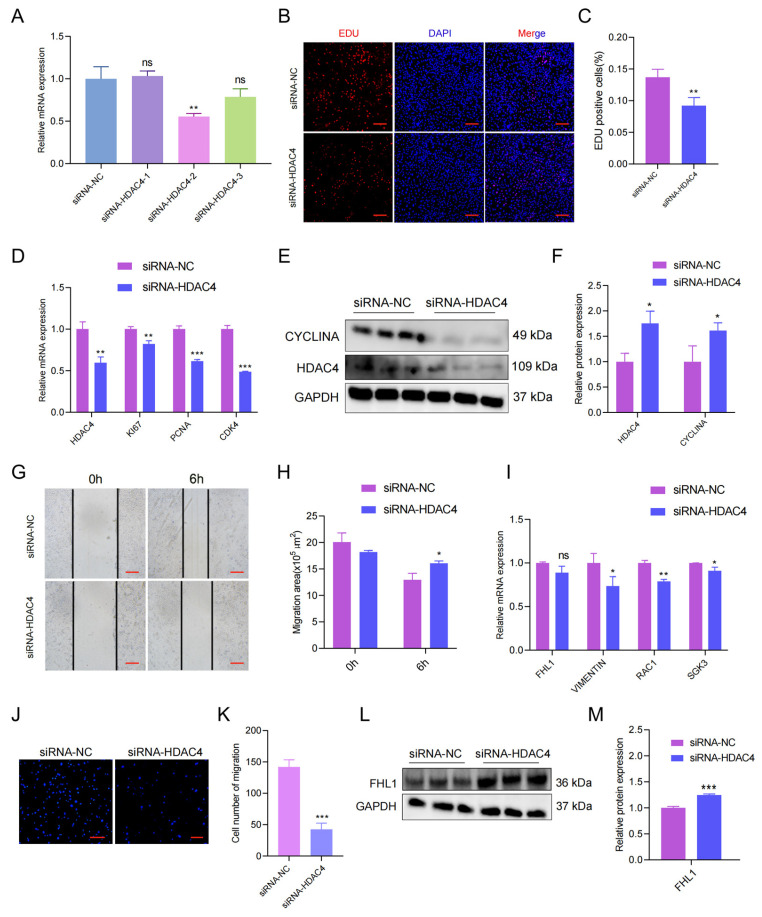
*HDAC4* regulates the proliferation and migration of porcine myoblasts. (**A**) Screening of the siRNA with the best knockdown effects. (**B**) The number of positive cells, and DNA replication cells with EdU staining (red) and nuclei with Hoechst staining (blue). (**C**) The number of EdU-positive cells were counted with Image J. (**D**) The mRNA expression of *KI67*, *PCNA*, and *CDK4* after *HDAC4* knockdown was tested and measured by RT-qPCR. (**E**) Protein levels after HDAC4 knockdown were measured by Western blot. (**F**) Protein gray-scale values were evaluated by the Image J method. (**G**) Effect of HDAC4 knockdown on wound healing at 6 h after scratch treatment (40×). (**H**) Changes in the scratch area of pig skeletal muscle cells. (**I**) The mRNA expression of *VIMENTIN*, *SGK3*, and *RAC1* after *HDAC4* knockdown was determined by RT-qPCR. (**J**) Knockdown of *HDAC4* reduced the number of porcine skeletal muscle cells that migrated in the transwell assay (40×). (**K**) Number of pig skeletal muscle cells migrated. (**L**) The protein level of FHL1 after HDAC4 knockdown was determined by Western blot. (**M**) Protein gray-scale values were calculated by Image J. All experiments were independently repeated at least three times and normalized using *GAPDH* or *β-actin*. Data were given as the mean ± S.E.M. * *p* < 0.05, ** *p* < 0.01, *** *p* < 0.001, ns (not significant).

To explore the functions of the HDAC4 gene in the proliferation and migration of porcine skeletal muscle cells, we designed three siRNAs of HDAC4, and the siRNA-2 with the highest knockdown efficiency was chosen for subsequent experiments (*p* < 0.01; Figure 5A). When it came to cell proliferation, the knockdown of HDAC4 reduced the number of EdU-positive myoblasts in the EdU assay (Figure 5B,C). The RT-qPCR results showed that mRNA expressions of marker genes (KI67, PCNA, and CYCLINA) for proliferation were decreased under HDAC4 knockdown (*p* < 0.001; Figure 5D). Consistently, Western blot results showed that the marker gene (CYCLINA) for cell proliferation was down-regulated under HDAC4 knockdown (*p* < 0.05; Figure 5E,F). Then, the migration of porcine skeletal muscle cell lines was validated under HADC4 knockdown by wound healing assay. The results observed that the migration area did shorten significantly after HDAC4 knockdown (Figure 5G,H). The expression of migration marker genes (FHL1, VIMENTIN, RAC1, and SGK3) at the mRNA level were down-regulated by HDAC4 knockdown (*p* < 0.01; Figure 5I). In addition, transwell experiments also suggested that knockdown of HDAC4 inhibited the membrane of cells through the pore (*p* < 0.001; Figure 5J,K). Similarly, Western blot results showed that the protein expression of migration marker genes was down-regulated under HDAC4 knockdown (*p* < 0.001; Figure 5L,M). 

**Figure 6:**
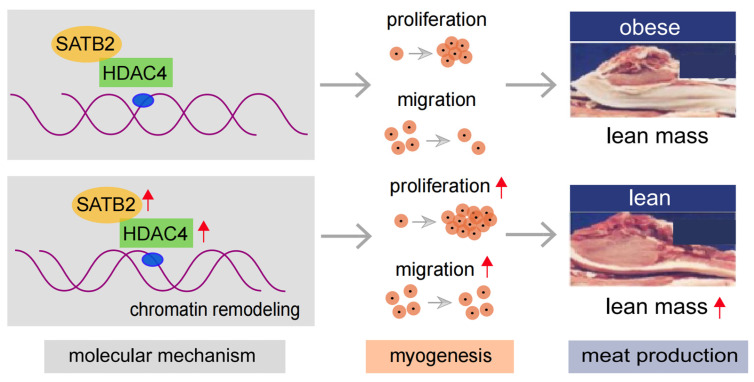
A cartoon illustration to show the regulatory mechanism of *SATB2* in myogenesis (pictures modified from our previous study [30]).

In addition, we also tested the other chromatin remodelers, including HDAC1, HDAC2, ARID1A, and INO80, and found no difference between Tongcheng and Landrace pigs (Figure S3A–D), and between Luchuan and Duroc pigs (Figure S3E,F). In summary, the results showed that SATB2 regulates the proliferation and migration of skeletal muscle cells via binding to HDAC4, which might mediate the chromatin organization to influence the myogenesis program (Figure 6).

## 4. Discussion

In this study, we aimed to explore the regulatory molecular mechanism of *SATB2* in porcine skeletal muscle cell fate concerning chromatin remodeling. As known, the skeletal muscle exerts a profound role in maintaining normal energy metabolism, physiological functions, and physical performance in pigs. According to the previous study, *SATB2*, as a crucial gene, exerts profound functions in the regulation of cortical development [17], neuron identity [31], colon stem cell identity [16], myoblast differentiation [20], and so on. This study characterized the *SATB2* from skeletal muscle at 27 developmental time points in obese- and lean-type pigs. Importantly, *SATB2* was selected by lean-type pigs compared to obese-type pigs [2]. In addition, *SATB2* holds a positive role in the regulation of skeletal muscle development.

When it comes to the functions of *SATB2*, our results indicated that *SATB2* promoted the proliferation and migration of porcine myoblasts. It is fitting that *SATB2* represses C2C12 cell differentiation [20]. Generally, muscle-controlled genes can promote C2C12 cell proliferation and migration, and inhibit the process of cell differentiation in myogenesis [31]. Similarly, *SATB2* can maintain the colonic identity of stem and differentiated cells [16]. Therefore, it indicated that *SATB2* played key roles in the process of myogenesis. 

Additionally, *SATB2* as a transcription factor might play crucial roles via the target gene. We explored and found one of the target genes, *HDAC4*, a chromatin remodeling factor related to myogenesis [32]. Our result was consistent with *SATB2* binding to *HDAC1* for neuronal differentiation [17]. Further, we confirmed the strong binding relationship between *SATB2* and *HDAC4*, even their consistent expression levels. Previous study also suggested that *SATB2* can bind to transcription factors *CDX2* and *HNF4A* to regulate colonic identity [16]. *SATB2* also can directly activate transcription of forebrain embryonic zinc finger 2 and SRY-box 5 for subcerebral neuron development [31]. We also indicated that *HDAC4* can induce the proliferation and migration of porcine skeletal muscle cells, which was consistent with *HDAC4* regulating satellite cell proliferation and differentiation and was a key factor for muscle reinnervation [33,34]. Thus, *SATB2* is a transcription regulator that orchestrates many aspects of physiological and molecular processes by regulating gene expression.

Epigenetic modifications are important for the growth and development of skeletal muscle, and the regulation mechanism of skeletal muscle satellite cells has been intensively studied [2,4]. Studies have revealed that chromatin remodeling plays an important role in the proliferation, migration, and differentiation of skeletal muscle cells [35]. As known, *SATB2* plays the biological regulatory functions as a chromatin organizer, which can bind to chromatin remodeling complexes and histone deacetylase to regulate chromatin organization, gene accessibility, and gene expression [15]. We found *SATB2* interacting with chromatin remodeling factor *HDAC4* to regulate myogenesis, which may medicate the chromatin structure change. Similarly, the loss of myoblast *SATB2* directly led to the alterations of chromatin folding, as evidenced by alterations of TAD and the reduction in chromatin loops [20]. A previous study demonstrated that *SATB2* was critical in shaping the chromatin environment and coordinating the myogenic differentiation program [17]. In addition, *SATB2* regulated the enhancer binding of *CDX2* and *HNF4A* [16]. Considerably, as a homolog of *SATB2*, *SATB1* can bind to specific DNA sequences, making the chromatin a ring-like structure, changing the distance of chromatin regions, and recruiting related functional proteins [36]. 

In this study, we also investigated some genes concerning the ATP-dependent chromatin remodeling complexes and histone post-translational modifiers [37,38]. However, we found that *ARID1A*, *INO80* (chromatin remodelers), and *HDAC1*, *HDAC2* (chromatin remodeling factors) displayed no significant difference between obese- and lean-type pigs. In terms of regulatory mechanisms, *SATB2* and *HDAC4* concerning chromatin remodeling may implicate the various chromatin accessibility and higher order chromatin structure (e.g., A/B compartment, TAD, and loop) of skeletal muscle cell in vitro [39]. In vivo in pigs, the changes of *SATB2* and *HDAC4* gene expression, even chromatin remodeling, may regulate physiological and metabolic processes and homeostasis, resulting in skeletal muscle growth, and development of meat production traits and individual health [4]. Taken together, this study only uncovered *SATB2* interacting with chromatin remodeling factor *HDAC4* in the process of myogenesis in pigs. However, our study was preliminary, the mechanism of *SATB2* via *HDAC4* regulating chromatin remodeling warrants further investigation of exploration and study. 

## 5. Conclusions

This study identified a key candidate gene *SATB2*, which acts in a crucial regulation role in skeletal muscle cell proliferation and migration. Mechanistic analyses showed that *SATB2* can bind to *HDAC4* to regulate skeletal muscle cell proliferation and migration, which might mediate the chromatin organization to influence myogenesis. This study provides a promising gene and new insights into understating the regulatory molecular mechanism of muscle myogenesis in pigs.

## Figures and Tables

**Table 1:** Information of the primers used in the RT-qPCR.

Gene Name	Primer Sequence (5′-3′)	Tm (°C)	Product Size (bp)
*β-actin*	F: GCGGCATCCACGAAACTAC	60	138
	R: TGATCTCCTTCTCATCCTGTC		
*SATB2*	F: ATGTCTATCACGTTGTGACGT	58	222
	R: ACTTGGTTGCTGATACGTGGC		
*KI67*	F: AGCCCGTATCTGGTGCAAAA	60	267
	R: CCTGCATCTGTGTAAGGGCA		
*PCNA*	F: AAGTCAAATCTGGTCTGTTAGCC	60	141
	R: CACTGTCCTGGGATGCTTGAA		
*CDK4*	F: TGGTTACAAGTGGTGGGACA	59	111
	R: CTGGAGCACGGTACCAGAGT		
*CYCLINA*	F: GCAGCAGCCTTTCATTTAGC	58	118
	R: GGTGAAGGTCCAGGAGACAA		
*CYCLIND2*	F: GTGCTGGGCAAGTTGAAGTG	60	158
	R: GCGAACTTGAAGTCAGTGGC		
*FHL1*	F: CCCCATCAACACCTTCCCTC	60	194
	R: ACACTCTCCCAATCGCCTTG		
*VIMENTIN*	F: TGTGAAGTGGATGCGCTCAA	58	268
	R: TTGGAAGAGGCAGAGAAATC		
*RAC1*	F: TCTTTTGAAAATGTTCGCGCA	58	205
	R: ACTCCAGGTATTTCACAGCAC		
*SGK3*	F: ACATCAACCTGGGACCATCT	55	113
	R: TAGTTTCCGTTTTGCAAGAA		
*HDAC4*	F: CCGTCTTTGCCCAACATCAC	60	248
	R: CCTGTGACCAGGGGTGTCT		

**Table 2:** The sequences of mutations in HDAC4.

Name	Sequence	Tm (°C)
pLV3-*HDAC4*-WT	F: CGAGCTCTTACGCGTGCTAGCTTTGTTAACCGCCGAGCGATGT	60
	R: ACTTAGATCGCAGATCTCGAGACCTAGTGAGGGAAGCACTG	
pLV3-*HDAC4*-MUT	F: TATAAAGTTTCATGGAAGCAGGCCGGTTCTAGAACCCTTT	60
	R: TGCTTCCATGAAACTTTATACCTCGC	

## Data Availability

The published datasets supporting the conclusions of this article are available in the Gene Expression Omnibus (GEO) at the National Center for Biotechnology Information (NCBI) under accession number SRP159036 and SRP304406, PRJNA740359 about pigs, and GSE185434 about mice.

## Supplementary Materials

The following supporting information can be downloaded at: https://www.mdpi.com/article/10.3390/genes15010065/s1, Figure S1: Flow cytometry to detect the cell cycle after transfection with (A) siRNA-NC and siRNA-SATB2, and (B) SATB2-NC and SATB2-OE; Figure S2: The HDAC4 characters of in the obese-type and lean-type pigs. (A) The sequences of mutation HDAC4. (B) HDAC4 characters various expression patterns among four development phases in the Landrace and Tongcheng pigs. (C) HDAC4 was higher expressed in the adult phases in the Landrace pig compared to the Tongcheng pig. (D) Correlation of HDAC4 with SATB2 in the adult phase of Landrace and Tongcheng pigs. (E) HDAC4 was higher expressed in the Duroc compared to Luchuan pigs. (F) Correlation of HDAC4 with SATB2 in Luchuan and Duroc pigs. Represents * *p* < 0.05, *** *p* < 0.001, ns (not significant); Figure S3: The chromatin remodelers in the obese-type and lean-type pigs. (A) HDAC1, (B) HDAC2, (C) ARID1A, and (D) INO80 in the Landrace and Tongcheng pigs. (E) HDAC1, HDAC2, and (F) ARID1A, INO80 in the Luchuan and Duroc pigs. Represents ns (not significant).
