# Supplementary material for: Transcription Factor SATB2 Regulates Skeletal Muscle Cell Proliferation and Migration via HDAC4 in Pigs
Source: Genes (Basel). 2024 Jan 2;15(1):65. doi: 10.3390/genes15010065 (PMC10815226; doi:10.3390/genes15010065)
Supplement: Supplementary file 1 [file genes-15-00065-s001.zip › genes-2796609-supplementary.pdf]

genes

MDPI

Article

Supplementary File

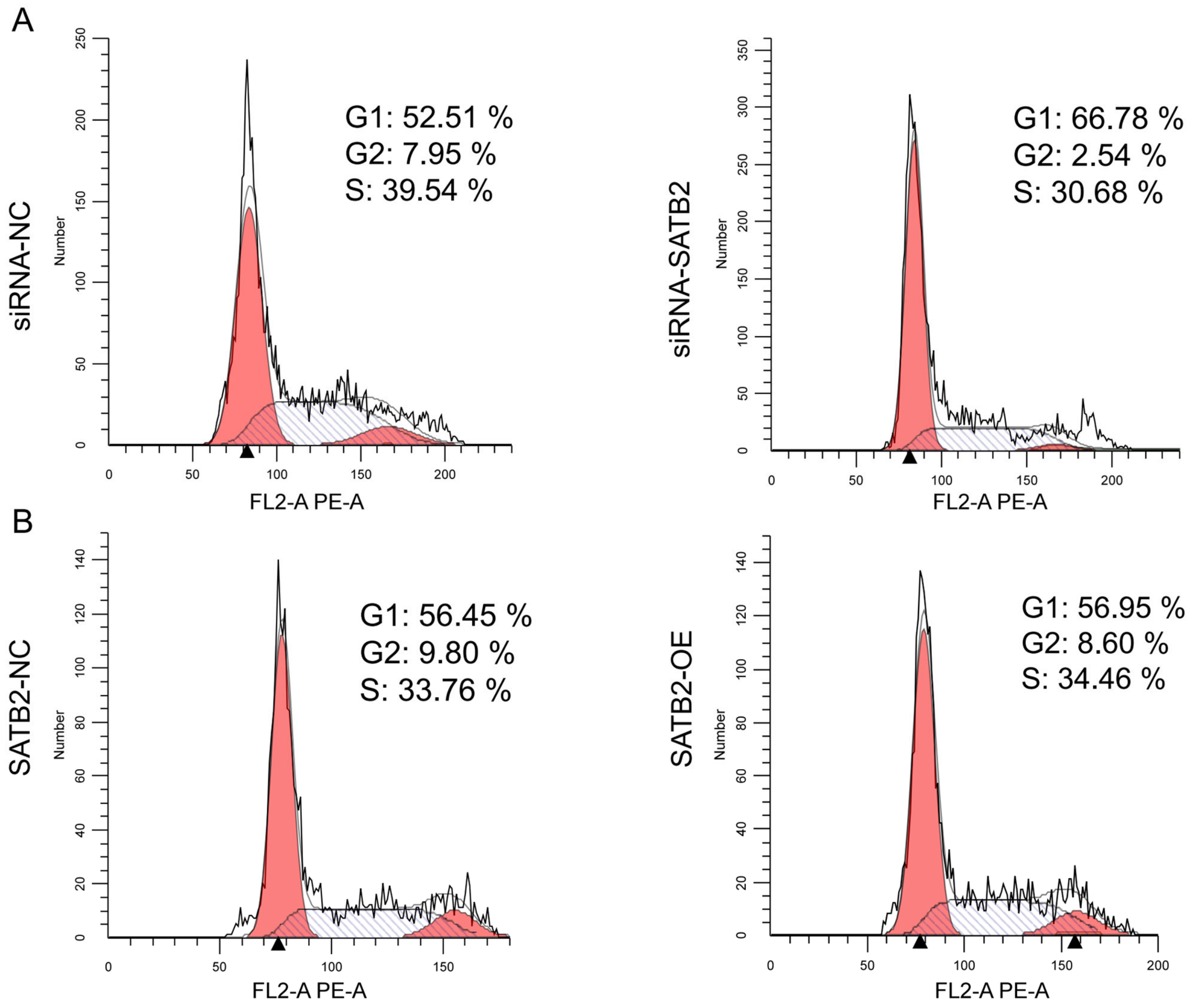

Figure S1. Flow cytometry to detect the cell cycle after transfection with (**A**) siRNA-NC and siRNA-SATB2, and (**B**) SATB2-NC and SATB2-OE.

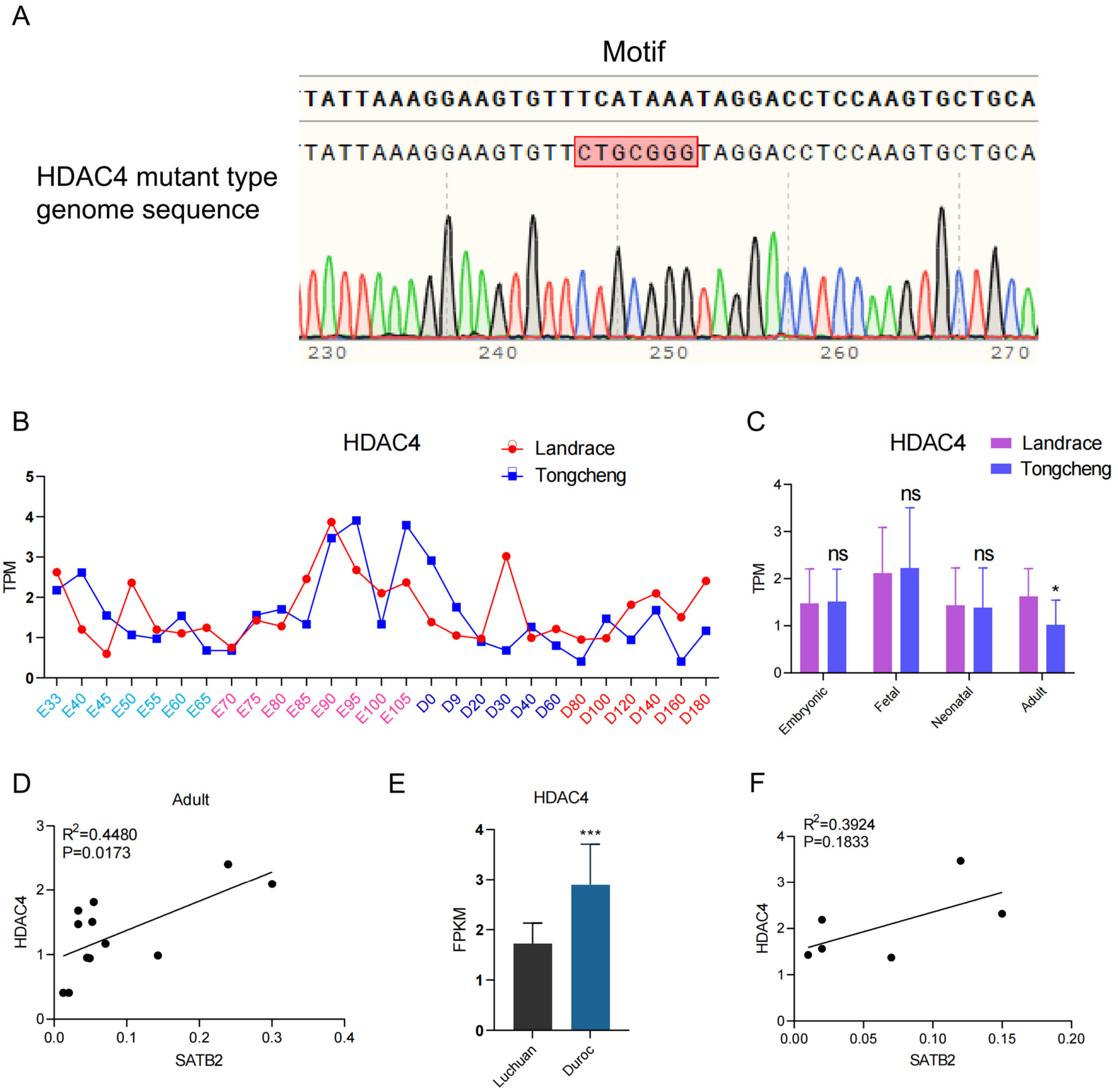

Figure S2. The HDAC4 characters of in the obese-type and lean-type pigs. (**A**) The sequences of mutation HDAC4. (**B**) HDAC4 characters various expression patterns among four development phases in the Landrace and Tongcheng pigs. (**C**) HDAC4 was higher expressed in the adult phases in the Landrace pig compared to the Tongcheng pig. (**D**) Correlation of HDAC4 with SATB2 in the adult phase of Landrace and Tongcheng pigs. (**E**) HDAC4 was higher expressed in the Duroc compared to Luchuan pigs. (**F**) Correlation of HDAC4 with SATB2 in Luchuan and Duroc pigs. Represents * $p < 0.05$, *** $p < 0.001$, ns (not significant).

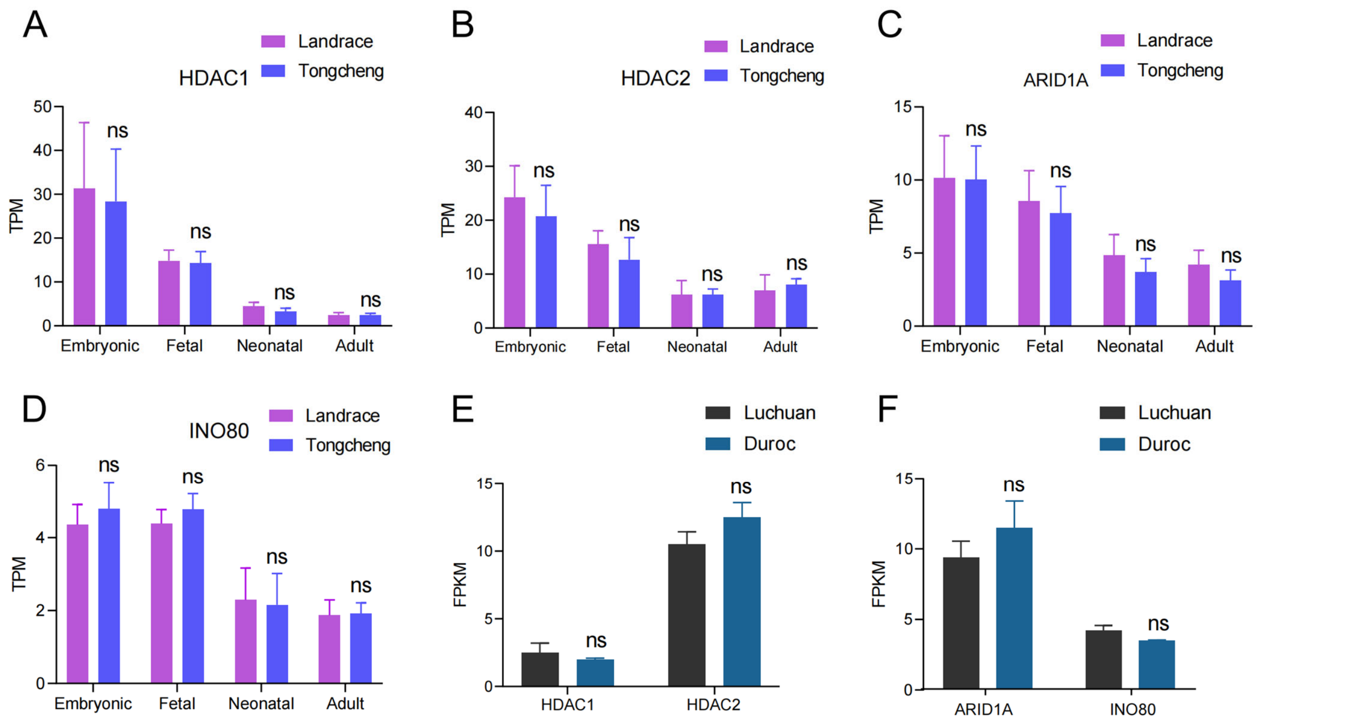

Figure S3. The chromatin remodelers in the obese-type and lean-type pigs. (**A**) HDAC1, (**B**) HDAC2, (**C**) ARID1A, and D INO80 in the Landrace and Tongcheng pigs. (**E**) HDAC1, HDAC2, and (**F**) ARID1A, INO80 in the Luchuan and Duroc pigs. Represents ns (not significant).
